# Targeting estrogen signaling and biosynthesis for aged skin repair

**DOI:** 10.3389/fphys.2023.1281071

**Published:** 2023-10-31

**Authors:** Helena D. Zomer, Paul S. Cooke

**Affiliations:** Department of Physiological Sciences, University of Florida, Gainesville, FL, United States

**Keywords:** 17β-estradiol, E2, wound healing, aging, older adults

## Abstract

Non-healing skin wounds are disproportionally prevalent in older adults. Current treatments do not account for the particularities of aged skin and result in inadequate outcomes. Overall, healing chronic wounds in the elderly remains a major unmet clinical need. Estrogens play a critical role in reproduction but also have important actions in non-reproductive organs. Estrogen biosynthesis and signaling pathways are locally activated during physiological wound healing, processes that are inhibited in elderly estrogen-deprived skin. Estrogen deprivation has been shown to be a critical mediator of impaired wound healing in both postmenopausal women and aged men, and topical estrogen application reverses age-associated delayed wound healing in both elderly men and women. These data indicate that adequate estrogen biosynthesis and properly regulated estrogen signaling pathways are essential for normal wound healing and can be targeted to optimize tissue repair in the elderly. However, due to fundamental questions regarding how to safely restore estrogen signaling locally in skin wounds, there are currently no therapeutic strategies addressing estrogen deficiency in elderly chronic wounds. This review discusses established and recent literature in this area and proposes the hypothesis that estrogen plays a pleiotropic role in skin aging and that targeting estrogen signaling and biosynthesis could promote skin repair in older adults.

## 1 Chronic skin wounds disproportionally affect older adults

Chronic wounds are skin wounds that fail to heal within a month. It is estimated that more than 3% of the American population over age 65 suffers from chronic wounds, a pattern seen worldwide that results in substantial morbidity and mortality for millions of people (Gould et al., 2015; Sen, 2021). Non-healing wounds develop more frequently and are directly associated with the presence of comorbidities, such as vascular diseases, chronic kidney disease, obesity, mobility issues, and diabetes, conditions that are more common in older adults. Most conditions associated with chronic, non-healing wounds have underlying metabolic causes (Sen, 2021). Overall, debilitating non-healing wounds disproportionally affect older adults, leading to physical restrictions, social isolation, negative emotions, psychological distress and loss of independence (Frykberg and Banks, 2015; Gould et al., 2015). Considering the worldwide growth of the aged population, problems affecting the elderly, such as chronic skin wounds, will become increasingly common (Gould et al., 2015). Therefore, there is a pressing need for better treatments to facilitate wound healing in older adults.

Although aging plays a major role in wound healing, it is often neglected in preclinical rodent models and clinical testing, resulting in a limited understanding of the mechanisms by which age slows healing (Gould et al., 2015). Current treatments for wound healing rely on a general approach based on cleaning, debridement, and dressing, a protocol that overlooks the particularities of aged skin and fails in 60% of the cases (Werdin et al., 2009; Huang et al., 2020). Ultimately, surgical interventions with autologous epidermal grafts may be used but these result in weak and thin skin with nearly a 35% failure rate (Reddy et al., 2014; Turissini et al., 2019; Berg et al., 2020; Gurtner et al., 2020). Novel leading experimental approaches use engineered materials and stem or skin cells to promote wound closure (Ho et al., 2017; Zomer et al., 2020). While outcomes are improved, clinical translation of strategies involving the transplantation of allogeneic and/or manipulated cells has been hampered due to safety concerns and the extended FDA regulatory approvals required. Overall, progress is needed in finding improved treatments for elderly skin repair. In order to be maximally effective, new treatments must address the underlying etiology of chronic wounds.

## 2 Estrogen is a pleiotropic factor in the hallmarks of aging

As a result of modern medicine and prolonged life expectancy, humans can spend more than a third of their lives in the post-reproductive stage, when sex steroid hormone levels markedly decrease (Howdon and Rice, 2018; Rzepecki et al., 2019; Čoma et al., 2021). Although it has long been known that concentrations of the most ubiquitous and important estrogen, 17β-estradiol (E2), decrease during the perimenopausal and menopausal period in women, a similar decline was recognized in men only recently (Finkelstein et al., 2013). Estrogen has since emerged as a critical determinant of aging in peripheral non-reproductive tissues, including bone, skin, and brain (Hardman and Ashcroft, 2008; Thornton, 2013; Seleit et al., 2016). Furthermore, age-associated estrogen deficiency is linked to several comorbidities, such as osteoporosis, dementia, and hearing loss (Manolagas, 2010; Park et al., 2019; Russell et al., 2019; Li et al., 2021). It is now well accepted that aging is intertwined with the effects of estrogen deficiency, with ovariectomized animals often used as a model of aging (Elliot et al., 2018).

Estrogen production peaks around 30 years of age in women, coinciding with the peak of skin collagen and elastin (Lephart, 2016; Lephart and Naftolin, 2021). Estrogen enhances blood flow and increases skin thickness, moisture, and hydration through the production of hyaluronic acid, mucopolysaccharides, and sebum (Nikolakis et al., 2016; Lephart and Naftolin, 2021). E2 also extends the anagen phase of the hair cycle, promoting hair growth by increasing the synthesis of essential growth factors that stimulate proliferation of hair follicle cells (Barakat et al., 2016). Moreover, estrogen has positive effects on the viability of skin fibroblasts, and by inducing the expression of transforming growth factor-beta (TGFβ) and tissue inhibitor of matrix metalloproteinases (TIMPs), and inhibiting matrix metalloproteinases (MMPs), E2 stimulates the production and maintenance of extracellular matrix components, such as collagen type I and III and fibrillin (Lephart and Naftolin, 2021).

The deleterious effects of estrogen deficiency on the skin are well-documented (Stevenson and Thornton, 2007; Rzepecki et al., 2019; Saville et al., 2019; Lephart and Naftolin, 2021; Wilkinson and Hardman, 2021). After menopause, collagen content decreases by 30% in the first 5 years and continues to drop by approximately 2% annually, resulting in skin that is thinner and weaker overall. Sebaceous and sweat gland function decreases, leading to drier, less hydrated skin, and loss of elastic fibers impairs skin pliability (Zouboulis and Boschnakow, 2001; Wilkinson and Hardman, 2020). Strikingly, estrogen deprivation was shown to be the principal mediator of impaired wound healing in the elderly population when a microarray study revealed that 78% of genes differentially expressed between wounds from young and aged men were estrogen-regulated, while only 3% were related to intrinsic aging (Hardman and Ashcroft, 2008; Emmerson and Hardman, 2012). Recent clinical studies have reported that women who undergo surgery during the pre-ovulatory phase, when estrogen levels are higher, show a decreased incidence of wound rupture and hypertrophic scarring regardless of age, race, and ethnicity (Lopez et al., 2016). Accordingly, women who receive hormone replacement therapy are less likely to develop venous leg ulcers or pressure ulcers than untreated controls, and anti-estrogen therapies, such as tamoxifen or aromatase inhibitors, are also correlated with poor wound healing (Thornton, 2013). Overall, extensive experimental evidence demonstrates that estrogen replacement therapies improve skin quality (thickness, elasticity, hydration), accelerate healing, and protect against the development of chronic wounds in aged humans and estrogen-deprived animal models (Ashcroft et al., 1997; Ashcroft et al., 1999; Kanda and Watanabe, 2003; Routley and Ashcroft, 2009; Emmerson and Hardman, 2012; Pomari et al., 2015; Brufani et al., 2017).

The mechanisms underlying these effects include modulation of the skin immune response by suppressing the production of inflammatory cytokines, such as interleukin (IL)-1 and tumor necrosis factor-alpha (TNFα), and increasing the production of anti-inflammatory cytokines, such as IL-4 and IL-10, therefore promoting a favorable environment for tissue healing and reducing inflammation (Routley and Ashcroft, 2009; Thornton, 2013). Anti-inflammatory effects were also reported by the downregulation of neutrophil L-selectin (SELL) and elastase (ELANE), which are associated with neutrophil accumulation and the development of chronic skin wounds (Ashcroft et al., 1999), and reduction of infiltrating macrophages and promotion of macrophage polarization to the M2 (pro-repair) phenotype, ultimately leading to an accelerated transition from the inflammatory to the proliferative phase of healing (Campbell et al., 2014; Brufani et al., 2017).

Estrogen was also shown to play a crucial role in controlling oxidative stress, a major contributor to impaired wound healing, by increasing the production of antioxidants, such as superoxide dismutase (SOD), and promoting B-cell lymphoma 2 (BCL2) expression, via activation of the nuclear factor erythroid 2-related factor (NRF2) (Kanda and Watanabe, 2003; Ambrozova et al., 2017; Song et al., 2019). Moreover, estrogen was reported to regulate proteolysis through the induction of protease inhibitors, preventing excessive degradation of extracellular matrix components that are essential for tissue repair (Ashcroft et al., 1999; Horng et al., 2017).

**FIGURE 1:**
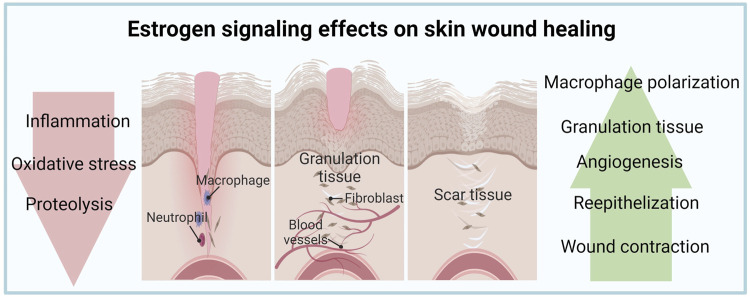
Estrogen signaling effects on skin wound healing. Estrogen has beneficial effects on inflammation, proliferation, and remodeling phases of skin wounds.

During the proliferative phase of healing, E2 induces the expression of pro-angiogenic factors such as VEGF and PDGFA in endothelial cells to promote angiogenesis (Toutain et al., 2009; El Mohtadi et al., 2021). Additionally, estrogen was found to stimulate epidermal and dermal cell migration and proliferation by enhancing the secretion of growth factors, such as epidermal growth factor (EGF) and TGFβ, aiding re-epithelialization and granulation tissue formation (Ashcroft et al., 1997; Pomari et al., 2015). At the remodeling stage, E2 modulates the secretion of extracellular matrix components and wound tensile strength (Lephart and Naftolin, 2021); however, the ultimate effect of E2 on scars is unclear. Novotny et al. (2011) demonstrated that E2 stimulates the differentiation of fibroblasts into myofibroblasts inducing wound contraction, and increases extracellular matrix deposition and wound tensile strength (Novotný et al., 2011). In contrast, earlier reports described lower tensile strength in ovariectomized rat wounds upon E2 treatment (Gál et al., 2010) and improved cosmetic appearance of scars (based on color, texture, and contour) of estrogen-deficient postmenopausal women associated with reduced extracellular matrix deposition (Ashcroft et al., 1997). Known mechanisms underlying estrogenic effects in skin repair are summarized in Table 1 and Figure 1.

**TABLE 1:** Described mechanisms of estrogen in skin wound healing. ↑↓ Arrows indicate an increase/decrease.

*Healing phase*	*Mechanism*	*Target*	*Receptor involved*	*Involved factors*	*Ref.*
*Inflammatory*	Macrophage polarization M1→M2	Macrophages	ERα	↑ ARG1, ↑ IL-10, ↓ iNOS, ↓ TNFα	Routley and Ashcroft (2009), Campbell et al. (2014), Brufani et al. (2017)
	↓ Inflammation	Neutrophils, macrophages	ERα	↓ MIF, ↑ IL-4, ↑ IL-10, ↓ SELL, ↓ TNFα, ↓ IL-1	Ashcroft et al. (1999), Routley and Ashcroft (2009), Thornton (2013)
	↓ Oxidative stress	Various	ERα	↑ SOD, ↑ NRF2, ↑ BCL2	Kanda and Watanabe (2003), Oh et al. (2019), Song et al. (2019)
	↓ Proteolysis	Extracellular matrix	ERα	↑ ARG1, ↑ TIMPs, ↓ ELANE, ↓ MMP2/8/9/13	Ashcroft et al. (1999), Horng et al. (2017)
*Proliferative*	↑ Granulation tissue	Fibroblasts	ERα	↑ TGFβ, ↑ FGF2, ↑ COL I/III, ↑ PDGFA	Shanker et al. (1995), Ashcroft et al. (1997)
	↑ Angiogenesis	Endothelial	ERα	↑ IL1β, ↑ VEGF, ↑ PDGFA	Emmerson and Hardman (2012), El Mohtadi et al. (2021)
	↑ Reepithelization	Keratinocytes	ERβ	↑ EGF, ↑ TGFβ, ↑ GM-CSF	Ashcroft et al. (1997), Campbell et al. (2010), Zhou et al. (2016)
*Remodeling*	↑ Wound contraction	Fibroblasts, extracellular matrix	ERα	↑ COL I/III, ↑ PDGFA	Shanker et al. (1995), Kanda and Watanabe (2005)

**FIGURE 2:**
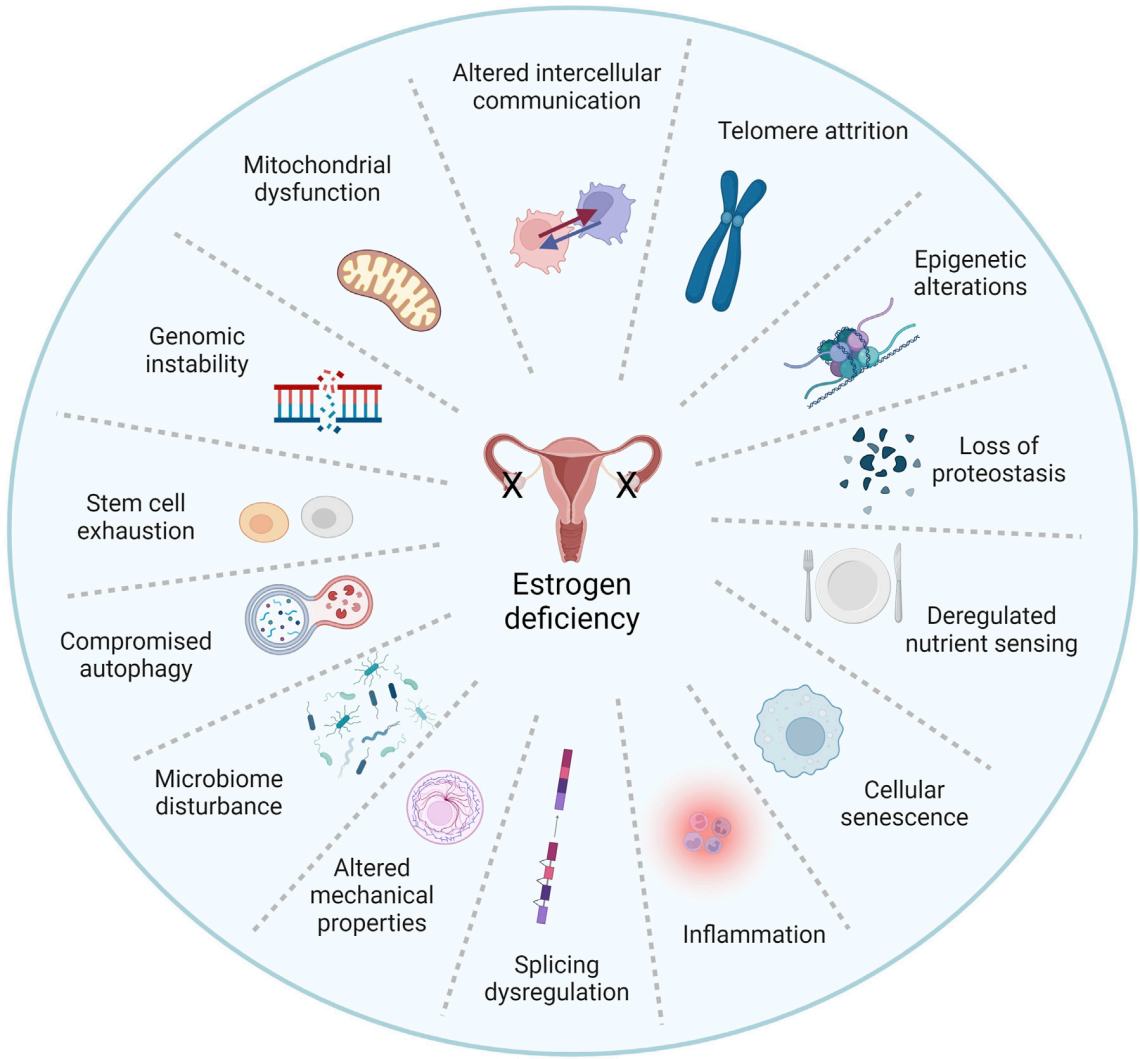
Estrogen is a pleiotropic factor in the hallmarks of aging. The pillars of aging as described by Kennedy et al. (2014) and Schmauk-Medina et al. (2022) are highly intertwined processes, and understanding the interplay between these factors is critical. Remarkably, existing literature has linked estrogen signaling to virtually all hallmarks of aging. Herein we propose that estrogen deficiency plays a major role in skin aging biology. Adapted from Schmauk-Medina et al. 2022, licensed under CC BY 3.0.

Beyond its effects on skin and wound healing, estrogen was shown to protect against multiple aspects of cellular aging. Estrogen treatment inhibits lipoperoxides and caspase 3 and 8 associated with oxidative stress and apoptosis in aged keratinocytes (Tresguerres et al., 2008). Estrogen also mitigates mitochondrial dysfunction, which is linked to age-related skin deterioration (Klinge, 2020). Studies also show that estrogen and phytoestrogens influence epigenetic modifications, preserving the integrity of the genome and reducing the accumulation of age-related changes (Kovács et al., 2020; Singh and Paramanik, 2022). Additionally, estrogen helps maintain telomere length by regulating telomerase activity, and inhibits cellular senescence, thus preserving cellular function and promoting skin rejuvenation (Figure 2) (Kanda and Watanabe, 2003; Bayne et al., 2008; Emmerson and Hardman, 2012; Kennedy et al., 2014; Oh et al., 2019; Schmauck-Medina et al., 2022; Taheri et al., 2022).

In addition to direct effects of E2, metabolites resulting from E2 biotransformation, such as catechol estrogens and methoxy estrogens have important biological activity in several different tissues (Park, 2018). In the vascular system, catechol estrogens regulate nitric oxide production and have other modulatory functions (Jobe et al., 2010; Landeros et al., 2019), which could impact the process of angiogenesis during wound healing. However, no effects of catechol estrogens in the skin have been described and the role of E2 metabolites during physiological and aged wound healing remains virtually unknown.

## 3 Estrogen signaling in skin

**FIGURE 3:**
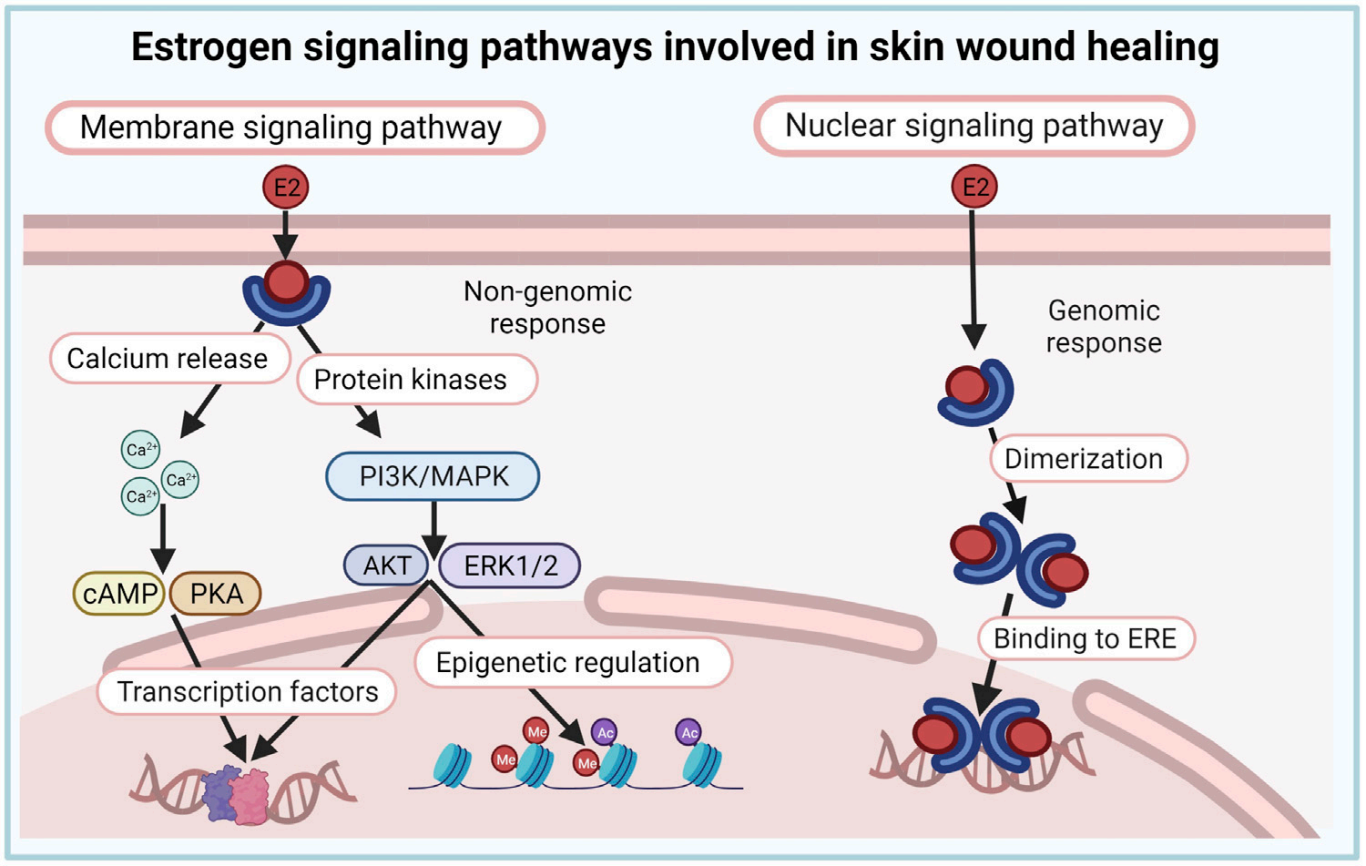
Estrogen signaling pathways involved in skin wound healing. Estrogen effects in the skin are likely mediated through both genomic (nuclear) and non-genomic (membrane) signaling pathways.

Estrogen actions are mediated through canonical nuclear and/or non-canonical membrane-initiated steroid signaling (Fuentes and Silveyra, 2019; Cooke et al., 2021). In the nuclear pathway, E2 binds either estrogen receptor (ER) α or ERβ in the cytoplasm, forming heterodimers and homodimers that translocate to the nucleus, bind DNA at estrogen response elements (ERE), and activate the expression of ERE-dependent genes. In the membrane signaling pathway, E2 binds to and exerts rapid actions through a subpopulation of ER located at the plasma membrane (mER) or G protein-coupled estrogen receptor (GPER). Activation of these membrane-associated receptors triggers various signaling pathways (e.g., increased Ca^++^, cAMP, or protein kinase cascades) that regulate downstream transcription and epigenetic factors (Fuentes and Silveyra, 2019; Cooke et al., 2021). Estrogen effects in the skin are likely mediated through both genomic (nuclear) and non-genomic (membrane) signaling pathways (Figure 3), although this has not been directly investigated, and data regarding the role and expression of ER in skin cells and during wound healing are inconsistent (Verdier-Sevrain et al., 2004).

Overall, most studies indicate that virtually all cells involved in skin repair (fibroblasts, keratinocytes, inflammatory cells, tissue stem cells, and vascular endothelial cells) express both ERα and ERβ and are direct targets of E2 (Matsubara and Matsubara, 2012; Emmerson et al., 2013; Pomari et al., 2015; Oh et al., 2019). Using an ERE-luciferase mouse, a reporter mouse that allows monitoring of ER transcriptional activity in a spatiotemporal manner, it was demonstrated that estrogen signaling is locally activated in keratinocytes *in vitro* (with 1 nM E2) and *in vivo* (with 10 nM E2). *In vivo*, E2 treatment also activated estrogen signaling in inflammatory cells at the wound site during skin wound healing, a process that was inhibited in estrogen-deprived skin and restored upon estrogen application (Emmerson et al., 2013; Brufani et al., 2017). Surprisingly, E2 promoted mouse fibroblast scratch wound closure in cell cultures but failed to induce ERE signal with doses ranging from 1 µM to 100 pM, suggesting that E2 effects on fibroblasts may be mediated through non-genomic pathways (Emmerson et al., 2013). In contrast, Pomari et al. found that E2 stimulated both human keratinocyte and fibroblast migration at early (4 h) and late (24–48 h) time points, suggesting the involvement of genomic and non-genomic signaling (Pomari et al., 2015). Of note, mouse dermal fibroblasts in unwounded skin expressed negligible luciferase and signal remained low in the first 3 days post-wounding. However, dermal fibroblasts are mostly active during the proliferative phase of healing, peaking at day 5–7 post-wounding, at time points that have not been investigated (Emmerson et al., 2013; Zomer and Trentin, 2018).

Both ERα and ERβ are critical for the healing process but their roles in wound healing differ. To address the role of local ERα and ERβ in the wound site, ovariectomized ERE-luciferase mice were treated with selective agonists for ERα, propylpyrazoletriol (PPT), and ERβ, diarylpropionitrile (DPN) (Emmerson et al., 2013). Administration of either compound during wound healing induced a luciferase signal in the peri-wound area, with a strong dermal cell signal. Both PPT and DPN showed anti-inflammatory effects in the skin, reducing neutrophil and macrophage influx to the granulation tissue; however, only DPN induced epidermal keratinocyte migration and accelerated wound closure, with a potency equivalent to E2 (Campbell et al., 2010). These studies highlighted that delayed healing in ovariectomized female mice can be reversed by stimulating ERβ signaling alone, and established ERβ activation as crucial in regulating estrogen’s beneficial skin effects, independent of estrogen’s anti-inflammatory properties (Campbell et al., 2010). In addition, induction of extracellular matrix deposition and enhanced tensile strength were shown to be mediated mainly through ERβ rather than ERα (Novotný et al., 2011). Accordingly, aging is associated with decreased ERβ expression in the epidermis and a reduced ability to efficiently repair injured skin (Inoue et al., 2011) and a polymorphism in the human ERβ gene is highly correlated with venous ulceration in the British Caucasian population (Ashworth et al., 2008). However, despite signaling through ERα alone failing to improve wound closure in ovariectomized mice, the beneficial effects of E2 on inflammation, angiogenesis, and wound contraction were reportedly mediated via ERα (Toutain et al., 2009; Novotný et al., 2011; Emmerson and Hardman, 2012).

Although most research has focused on genomic signaling by the main ERs, ERα and ERβ, participation of non-canonical pathways and other receptors have also been demonstrated (Campbell et al., 2010; Campbell et al., 2014; Zhou et al., 2016; Horng et al., 2017). Estrogen was shown to accelerate skin wound healing by promoting keratinocyte proliferation via non-genomic ERK/AKT signaling (Zhou et al., 2016). Additionally, E2 is known to be able to signal through the insulin-like growth factor 1 receptor (IGF-1R) in the skin in a non-genomic manner and this process has been implicated in the regulation of cutaneous aging (Emmerson and Hardman, 2012). There is also increasing evidence suggesting that rapid, non-genomic estrogen signaling is mediated by GPER in the skin (Serizawa et al., 2017). However, specific roles of E2 signaling pathways during skin homeostasis and repair remain to be fully elucidated.

## 4 Skin estrogen biosynthesis

Despite the gonads being the primary source of androgens and estrogens, active sex steroids are also synthesized locally in peripheral tissues from circulating precursors (Pomari et al., 2015). Before menopause, human estrogen biosynthesis occurs largely in the ovary and the testis, adipose tissue, brain, adrenals, skin, and bones (Taves et al., 2011; Slominski et al., 2013; Nikolakis et al., 2016). The human skin is not only an estrogen target but also a primary estrogen source; however, this has not been reported in mice (Pomari et al., 2015).

The human skin is a complete steroidogenic organ, as intracrine estrogen biosynthesis occurs either from precursors of adrenal origin (dehydroepiandrosterone sulfate [DHEA-S] and DHEA) or through the conversion of cholesterol to pregnenolone and its subsequent transformation to biologically active estrogen in skin cells (Labrie et al., 2000; Pomari et al., 2015). Postmenopausally, estrogen produced in the skin from the conversion of the adrenal precursors DHEA-S and DHEA becomes the primary source of estrogen; however, overall steroidogenesis is reduced with aging in both sexes (Labrie et al., 2000). Steroid production in the skin is directly dependent on local enzymatic activity (e.g., aromatase), substrate availability, and mobilization of signal transduction and gene expression pathways, and can be modulated by several internal and external factors, including growth factors, inflammatory cytokines such as IL-1β and TNFα, cAMP analogs, and glucocorticoids such as dexamethasone (Tkachenko et al., 2011; Inoue et al., 2012; Slominski et al., 2013; Pomari et al., 2015; Barakat et al., 2016).

Keratinocytes, melanocytes, and dermal fibroblasts express the key steroidogenic enzymes required for estrogen biosynthesis (Pomari et al., 2015; Lephart and Naftolin, 2021). Additionally, strong aromatase expression has also been shown in both hair follicles and sebaceous glands (Slominski and Wortsman, 2000; Barakat et al., 2016). Wounding leads to changes in aromatase activity in skin cells; while aromatase expression is increased in epidermal keratinocytes 24 h after mechanical wounding, it is decreased in dermal fibroblasts (Pomari et al., 2015). These changes result in an overall amplification of intracellular estrogen bioavailability and support a mechanism by which local concentrations of estrogen can change quickly following acute stress or damage (Pomari et al., 2015). Skin steroidogenic potential and production are limited, so skin-derived estrogens play site-specific autocrine and/or paracrine regulatory roles (Taves et al., 2011; Slominski et al., 2013). Since skin steroidogenesis can be modulated by external factors and is locally restricted, there is a tremendous potential for novel topical therapeutic approaches targeting skin estrogen biosynthesis to achieve local estrogen healing benefits without off-target effects which are undesirable in both men and women, as explained in the following section.

## 5 Limitations of current estrogen-based therapies

**FIGURE 4:**
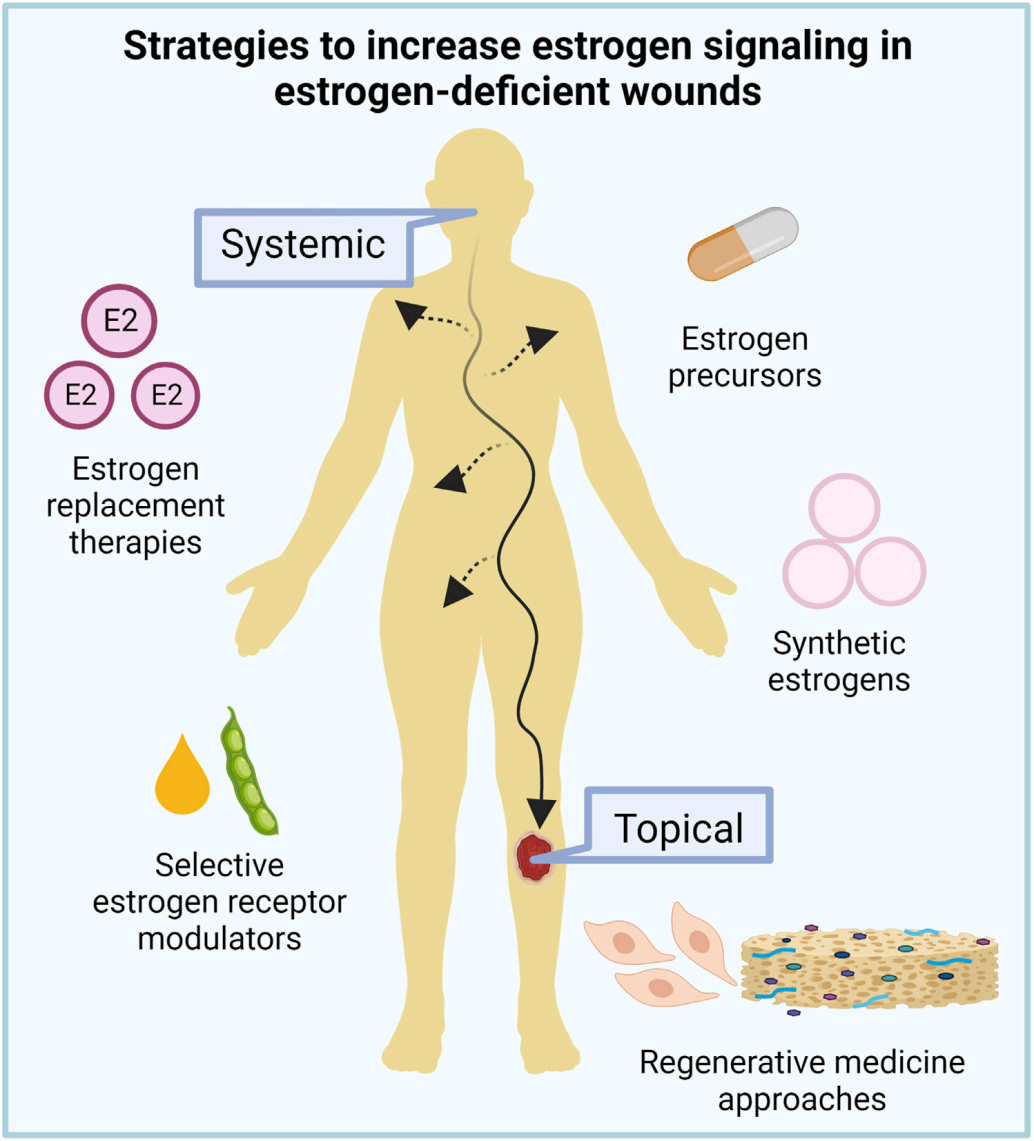
Targeting estrogen signaling for aged skin repair. Given the deleterious side effects of current estrogen replacement therapies, new systemic and topical strategies to increase estrogen signaling in estrogen-deficient wounds are under investigation to improve aged skin repair.

Traditional systemic estrogen replacement therapies, with doses varying from 150 to 2000 µg/day are effective for treating menopausal symptoms such as hot flashes and vaginal dryness and also have beneficial effects on bone loss, and skin appearance and repair. However, despite the clear benefits of estrogen replacement therapy on the skin, its use remains controversial. Deleterious off-target effects in women 10 years or more past menopause, such as ovarian, endometrial, and breast cancers, intravascular thrombosis, cardiovascular episodes, and stroke, and suppression of endogenous testosterone and libido and increased risk of prostate cancer in men, have precluded broad clinical applications (Anderson et al., 2004; Hall and Phillips, 2005; Stevenson and Thornton, 2007). These negative effects are mostly associated with the systemic activation of ERα signaling (Ahn et al., 2020); therefore, current research efforts have focused on novel strategies that can act locally rather than systemically in the skin and/or can selectively activate ERβ to safely promote wound healing in estrogen-deficient patients (Figure 4).

## 6 Novel strategies targeting estrogen signaling and/or biosynthesis for skin wound healing

### 6.1 Selective estrogen receptor modulators

Recent studies have tested natural and engineered selective estrogen receptor modulators (SERMs) to increase estrogen signaling in non-healing wounds of elderly patients without systemic side effects (Stevenson and Thornton, 2007; Čoma et al., 2021). Tamoxifen and raloxifene are currently the most commonly used SERMs. Both induce tissue-selective ER agonist or antagonist effects depending on the interaction with tissue-selective transcriptional coregulators that are recruited by each ligand-specific receptor conformation (McDonnell et al., 2021). Tamoxifen has been clinically used in ERα-positive breast cancers as an antagonist of estrogen signaling in mammary epithelial cells; simultaneously, it elicits secondary ERα-agonist effects in bone, preventing osteoporosis, but also acts as an agonist in the uterus, leading to an increased risk of endometrial cancer (Braun et al., 2016; Rachner et al., 2018; Emons et al., 2020). Both tamoxifen and raloxifene were shown to promote anti-inflammatory effects in macrophages, stimulate fibroblast proliferation, and inhibit myofibroblast differentiation *in vitro* (Stevenson et al., 2009; Carthy et al., 2015) and accelerate wound closure in ovariectomized mice (Hardman et al., 2008). However, overall knowledge of the activity of these SERMS in the skin is still limited.

Phytoestrogens are non-steroidal compounds derived from plants that are structurally and functionally similar to E2 (Ososki and Kennelly, 2003). The soy-derived genistein (4′,5,7-trihydroxyisoflavone) displays SERM characteristics and represents a promising natural alternative source of biologically active compounds with estrogenic activities. Genistein has a higher binding affinity for ERβ than ERα and its activity depends on endogenous estrogen levels and ER expression (Irrera et al., 2017; Čoma et al., 2021). It has the unique ability to selectively act as an agonist or antagonist in a dose- and tissue-specific manner and has been shown to improve skin repair while exerting anti-neoplastic and chemopreventive properties in both *in vitro* and in rodent models (Emmerson et al., 2010; Marini et al., 2010; Park et al., 2011; Pawlicka et al., 2022; Peng et al., 2023). Genistein was shown to modulate angiogenesis in human and mouse endothelial cells and induce macrophage polarization to a pro-repair phenotype, two important processes during physiological wound healing (Abron et al., 2018; Berndt et al., 2018; Peng et al., 2023). Accordingly, genistein reduced inflammation and oxidative stress in early-stage diabetic mice wounds and accelerated wound closure and improved tensile strength in ovariectomized mouse wounds (Emmerson et al., 2010; Marini et al., 2010; Eo et al., 2016).

Genistein’s effects on the skin could also aid older adults’ wound healing by delaying skin aging. *In vitro* studies suggested that genistein treatment of human keratinocytes and fibroblasts modulates glutathione content and reactive oxygen species release, endothelial/inducible (e/i)NOS dependent nitric oxide release, MMP expression, and mitochondria membrane potential through mechanisms involving p38 MAPK, AKT and ERK1/2 as downstream signaling associated with membrane ERs and GPER, as well as by increasing SOD activity and BCL2 expression in endothelial cells (Savoia et al., 2018; Čoma et al., 2021).

Although SERMs are a promising approach to treating estrogen-deficient skin wounds given that they can selectively target estrogen receptors in specific tissues, this also means that their effects may be limited to specific cell types or tissues. This specificity may not address the multifactorial nature of wound healing, which involves multiple cell types and complex interactions. Additionally, long-term effects and potential risks associated with prolonged SERM use in wound healing applications are still not well understood. The possible interaction of SERMs with endogenous estrogen and signaling pathways will also need to be investigated, as the interplay between exogenous SERMs and endogenous estrogen levels needs to be carefully considered to ensure optimal therapeutic effects. Finally, although *in vitro* and preclinical studies have provided promising results, clinical evidence supporting the use of SERMs in skin wound healing is still limited. There is a need for well-designed clinical trials to evaluate the efficacy, safety, and optimal dosing strategies of SERMs specifically for wound healing purposes.

### 6.2 Estrogen precursors

The estrogen precursor DHEA(-S) is a ubiquitous adrenal hormone generally considered to be a weak androgen; however, it is also a precursor for the intracrine biosynthesis of potent androgens and estrogens in peripheral tissues that express the relevant steroidogenic enzymes, such as the skin (Labrie et al., 2003; Mills et al., 2005). It is suggested that high levels of DHEA have a beneficial effect on longevity and prevent several human pathologic conditions, including heart disease, diabetes, and chronic venous leg ulcers (Legrain and Girard, 2003; Schwartz and Pashko, 2004; Mills et al., 2005; Pomari et al., 2015). In adult women, DHEA-S is the most abundant steroid, circulating at micromolar concentrations up to 10,000 higher than that of E2 (Labrie et al., 2000). However, circulating DHEA and DHEA-S levels decline with aging and are reduced to only 5%–20% of their peak values in individuals past age 70 (Labrie et al., 1995). Because DHEA is only converted into sex steroids in target tissues containing the relevant physiologic enzymatic machinery, it has been suggested that it could be used for treating estrogen-deficient wound healing without deleterious systemic side effects (Pomari et al., 2015). *In vitro* studies showed that DHEA and DHEA-S accelerated dermal fibroblast migration (Stevenson et al., 2009) and prevented keratinocyte apoptosis via membrane binding sites (Alexaki et al., 2009), and these processes were reversed when inhibitors of conversion enzymes (aromatase or STS) were used, confirming that conversion to estrogen is required (Pomari et al., 2015). Conversely, DHEA inhibited inflammation and accelerated skin wound healing in an ER-dependent manner in ovariectomized and aged male mice models (Mills et al., 2005). These studies, although limited, indicate that DHEA and DHEA-S may represent a viable strategy to treat elderly estrogen-deprived skin wounds.

### 6.3 Synthetic estrogens

In an effort to create estrogenic molecules intended for direct skin application without causing systemic effects, Brufani et al. (2009, 2017) synthesized E2 derivative compounds that rapidly undergo metabolic oxidation and lose their affinity for ERs, limiting their effects (Brufani et al., 2009; Brufani et al., 2017). When administered to a wound in an ovariectomized ERE-luciferase mouse model, these compounds locally activated ERE without triggering any systemic effects (Brufani et al., 2017). Notably, the researchers observed reduced inflammation and enhanced wound healing effects comparable to the E2 treatment used as a positive control (Brufani et al., 2017). Further development of such approach could help pave the way for the development of novel drugs to address estrogen-deficient wounds.

### 6.4 Regenerative medicine approaches

Given the complexity of wound healing, particularly in older adults with multiple morbidities, and the progressive, degenerative impact of estrogen deficiency on structural components of skin, optimal healing strategies must be able to induce regenerative processes to properly restore skin form and function. Although there is vast research on regenerative medicine approaches for skin wound healing, including cellular and biomaterial-based strategies, most of them are focused on young rather than aged subjects and the impact of estrogen deficiency in the outcomes of such therapies is still largely unknown.

Cellular therapies have emerged as a promising strategy in regenerative medicine for skin wound healing. Mesenchymal stem/stromal cells (MSCs) are multipotent stem cells present in virtually all adult tissues (Dominici et al., 2006; Meirelles et al., 2006). Their ability to successfully accelerate and improve the quality of skin wound healing has been widely demonstrated in animal studies and clinical trials (Wu et al., 2007; Clover et al., 2014; Formigli et al., 2015; Zomer et al., 2020; Kerstan et al., 2022). MSCs secrete multiple cytokines and growth factors with anti-inflammatory, angiogenic, proliferative, migratory and anti-apoptotic effects which have been linked to their broad therapeutic application (Jo et al., 2021). It has been shown that MSCs increase tensile strength through a macrophage-dependent mechanism in wound healing in old mice, restoring the strength levels seen in younger adult mice (Lee et al., 2013). Additionally, MSCs and their secreted factors were shown to induce skin rejuvenation in human dermal fibroblasts by promoting autophagy and inhibiting senescence (Kim et al., 2018; Li et al., 2022). Strikingly, a recent study reported that rodent MSCs secrete E2 *in vitro* and *in vivo* and suggested that E2 secretion by MSCs is responsible for their therapeutic potential (Li et al., 2015). However, endogenous E2 levels were shown to interfere with MSC activity and cells isolated from aged or estrogen-deficient individuals showed impaired functions (Yao et al., 2016; Zhuge et al., 2018; Delben et al., 2021; Elliot et al., 2022). The complex crosstalk between E2 and MSCs needs to be further investigated.

The placenta is a highly specialized endocrine organ that supports normal fetal growth and development (Costa, 2016). Since embryo morphogenesis has many similarities to tissue repair, placental tissues have been extensively investigated as therapeutics for wound healing (Hong et al., 2010; Akela et al., 2013; Choi et al., 2013; Brigido et al., 2018; Wang et al., 2021). Accordingly, several *in vitro*, pre-clinical and clinical studies have reported low immunogenicity, anti-inflammatory, anti-bacterial, anti-oxidative, and anti-scarring properties of placental-derived materials in different skin wound healing models, and 28 decellularized or devitalized placental matrices are currently FDA approved for clinical use as wound dressings (Choi et al., 2013; Kim et al., 2015; Moore et al., 2015; Goswami et al., 2017; Heo et al., 2018). Strikingly, although the placenta is an important site of estrogen biosynthesis and a rich source of estrogens and estrogen signaling factors, potential estrogenic properties of placental materials remain virtually unexplored (Kong et al., 2008; Choi et al., 2014; Lee et al., 2020). Most studies using placental materials did not characterize their estrogen content; however, placentally-derived compounds alleviated postmenopausal symptoms in estrogen-deprived women, and clinical case reports described premature sexual development in children using placental-derived hair products, indicating that placentally-derived materials preserve some of the native tissue estrogenic effects (Tiwary, 1998; Phillips et al., 2008; Koike et al., 2012; Koike et al., 2013; Lee et al., 2020). These studies highlight the crucial requirement to rigorously characterize the estrogenic potential of placenta-derived materials to avoid undesirable effects but also emphasize that placental materials may be particularly beneficial to treat skin wound healing in older adults.

Recently, engineered biomaterials have been specifically developed as delivery systems for the targeted and controlled delivery of estrogen or estrogenic compounds to the wound site. The combination of diverse nanofibers with E2 or genistein from soy and other plants was shown to promote keratinocyte and fibroblast proliferation, cellular migration and infiltration, and integrin β1 expression *in vitro* (Ahn et al., 2018; Ahn et al., 2019). When applied to ovariectomized mouse and *ex-vivo* human wound models, these materials accelerated re-epithelialization and epidermal thinning and reduced scar formation, therefore promoting improved skin repair vs. controls (Unnithan et al., 2015; Ahn et al., 2018; Ahn et al., 2019; Ahn et al., 2020; Ramos‐rodriguez et al., 2021). These beneficial effects were dose-dependent and were abrogated when the ERβ signaling pathway was inhibited, but off-target systemic side effects have not been investigated. Although promising, studies in this area are still in the early phase.

## 7 Final remarks

Recognizing the importance of estrogen in skin homeostasis and repair presents an opportunity to develop innovative therapies that harness estrogen’s beneficial effects while minimizing systemic risks. The mechanisms by which estrogen promotes wound healing involve modulation of inflammation, oxidative stress, proteolysis, cell migration, proliferation, and angiogenesis, processes that were shown to be improved with the use of topical SERMs, estrogen precursors like DHEA, synthetic estrogens, and regenerative medicine approaches targeting estrogen signaling and biosynthesis. Although these strategies have the potential to enhance wound healing in older adults, they must be further investigated to ensure their efficacy, safety, and feasibility for clinical translation. Addressing the unique challenges posed by chronic wounds in the aged population will require interdisciplinary collaboration to advance our understanding of skin aging and wound healing mechanisms. Future research should focus on refining strategies that target estrogen pathways specifically in the skin, considering both genomic and non-genomic routes, to promote effective and safe wound healing interventions for older adults.

## Abbreviations

Arg1, Arginase 1; BCL2, B-cell lymphoma 2; Col I/III, Collagen type I and III; DHEA, Dehydroepiandrosterone; DHEA-S, Dehydroepiandrosterone sulfate; DPN, Diarylpropionitrile; E2, 17β-estradiol; EGF, Epidermal growth factor; ER, Estrogen receptor; ERE, Estrogen response element; ERK, Extracellular signal-regulated kinase 1/2; FDA, Food and Drug Administration; FGF2, Fibroblast growth factor 2; GPER, G-Protein-coupled estrogen receptor; GM-CSF, Granulocyte-macrophage colony-stimulating factor; IL, Interleukin; iNOS, Inducible nitric oxide synthase; MAPK, Mitogen-activated protein kinase; MIF, Macrophage migration inhibitory factor; MMPs, Matrix metalloproteinases; MSCs, Mesenchymal stem/stromal cells; NRF2, Nuclear factor erythroid 2-related factor; PDGFA, Platelet-derived growth factor A; PPT, Propylpyrazoletriol; SERMs, Selective estrogen receptor modulators; SOD, Superoxide dismutase; TGFβ, Transforming growth factor-beta; TNFα, Tumor necrosis factor-alpha; TIMPs, Tissue inhibitors of metalloproteinases; VEGF, Vascular endothelial growth factor.
